# What a Record

**Published:** 1883-06

**Authors:** 


### WHAT A RECORD.

A well-informed author estimates the number of teeth which are annually extracted in the United States, at twenty millions, and the number of artificial teeth used in the same period of time at eight millions.—*Zahntechnische Reform.*

